# Chitosan–Hydrazone-Modified Calcium Phosphate Scaffolds: Fabrication, Characterization, and Drug Delivery Potential

**DOI:** 10.3390/biomedicines13092270

**Published:** 2025-09-15

**Authors:** Teodora Jakovljević, Jelena Stanisavljević, Julijana Stevanović, Miloš Petković, Ivana Z. Matić, Miloš Papić, Suzana Živanović, Tamara Matić, Vukašin Ugrinović, Djordje Janaćković, Biljana Ljujić, Djordje Veljović

**Affiliations:** 1 Innovation Center of the Faculty of Technology and Metallurgy Ltd., University of Belgrade, Karnegijeva 4, 11000 Belgrade, Serbia; tjakovljevic@tmf.bg.ac.rs; 2 Faculty of Technology and Metallurgy, University of Belgrade, Karnegijeva 4, 11000 Belgrade, Serbia; jelena.stanisavljevic@etu.u-paris.fr (J.S.); tmatic@tmf.bg.ac.rs (T.M.); nht@tmf.bg.ac.rs (D.J.); 3 UFR Sciences Fondamentales et Biomédicales, Université Paris Cité, 45 Rue des Saijel-Pères, 75006 Paris, France; 4 Vinča Institute of Nuclear Sciences—National Institute of the Republic of Serbia, University of Belgrade, 11351 Belgrade, Serbia; julijana.tadic@gmail.com; 5 Department of Organic Chemistry, Faculty of Pharmacy, University of Belgrade, Vojvode Stepe 450, 11221 Belgrade, Serbia; milos.petkovic@pharmacy.bg.ac.rs; 6 Institute of Oncology and Radiology of Serbia, Pasterova 14, 11000 Belgrade, Serbia; ivanamatic2103@gmail.com; 7 Faculty of Medical Sciences, University of Kragujevac, Svetozara Markovića 69, 34000 Kragujevac, Serbia; milos.papic@fmn.kg.ac.rs (M.P.); suzanazivanovic91@yahoo.com (S.Ž.);

**Keywords:** calcium phosphate, chitosan, scaffold, hydrazone, biomedical application

## Abstract

**Background/Objectives**: Recent advancements in biomaterials aimed at closely mimicking natural biological tissues hold great promise for hard tissue regeneration and controlled drug release due to their superior physical, chemical, and biological properties. This study aimed to develop multi-ion doped calcium hydroxyapatite (HAp) scaffolds with chitosan-based coatings for localized drug delivery, incorporating a novel hydrazone compound with potential anticancer activity. **Methods**: HAp powders doped with magnesium (Mg^2+^), strontium (Sr^2+^), and varying fluoride (F^−^) contents (0–2 mol.%) were synthesized via a hydrothermal method. Scaffolds were fabricated using the sponge replica technique and subsequently coated with chitosan or a chitosan–hydrazone blend. Dopant incorporation was confirmed by electron dispersive X-ray spectroscopy (EDS). Phase composition and morphology were analyzed via X-ray diffraction (XRD) and scanning electron microscopy (SEM). Mechanical properties, bioactivity, cytotoxicity, and hydrazone release profiles were systematically evaluated. **Results**: EDS confirmed successful incorporation of Mg^2+^ and Sr^2+^ in all powders, while F^−^ was detected only in powders with 1 and 2 mol.% fluoride. XRD and SEM revealed the phase composition and scaffold microstructure. Chitosan coatings significantly improved scaffold compressive strength and reduced degradation rate, indicating enhanced stability in biological environments. The coated scaffolds supported MRC-5 fibroblast viability. The hydrazone compound exhibited dose-dependent antitumor cytotoxicity comparable to cisplatin and showed sustained release from scaffolds for up to 15 days. **Conclusions**: The combination of multi-ion doped HAp scaffolds and chitosan–hydrazone coatings provides a promising platform for bone tissue engineering and localized cancer therapy, demonstrating both mechanical stability and controlled, sustained drug release.

## 1. Introduction

In the fields of tissue engineering and regenerative medicine, there is an increasing demand for innovative biomaterials that offer improved biocompatibility, optimal mechanical properties, and enhanced biological functionalities [1]. These materials are essential for addressing the complex requirements of various biomedical applications. Modern biomaterials enhance integration with biological tissues, promoting optimal healing processes while also functioning as effective active substances carriers that enable controlled release of therapeutic agents at the site of injury or disease [2]. As research advances, the focus is shifting toward the development of bioactive materials that not only replicate the natural tissue environment but also actively support cellular activities and interactions essential for tissue regeneration and targeted active substances delivery [3].

Bioceramic materials based on calcium hydroxyapatite (HAp) play an important role in the fabrication of implants for use in orthopedics, in dentistry, in bone tissue engineering, and in systems for drug delivery of bioactive factors, or proteins, due to their bioactive properties, chemical composition, excellent biocompatibility, good mechanical properties, and structural similarity to bone and tooth minerals [4,5].

Natural hard tissues predominantly consist of HAp crystals as the main inorganic component [6]. HAp exhibits the ability to undergo partial substitution of calcium ions (Ca^2+^) with various cations, such as magnesium (Mg^2+^), strontium (Sr^2+^), manganese (Mn^2+^), copper (Cu^2+^), zinc (Zn^2+^), and silver (Ag^+^) [7,8,9,10], as well as the incorporation of anions like carbonate (CO_3_^2−^), fluoride (F^−^), and chloride (Cl^−^) in place of phosphate (PO_4_^3−^) or hydroxyl (OH^−^) ions [11]. The incorporation of specific cations and anions during the synthesis of calcium phosphate materials can significantly influence their properties [12].

Mg^2+^ ions, the most common substitutes for Ca^2+^, readily incorporate into the crystalline structure of HAp due to their chemical similarity [13]. Mg^2+^ ions are known to enhance the mineralization of bones and teeth, promote cell proliferation, and improve the bioactivity and biocompatibility of the material, aligning its mechanical and biological properties with those of natural biological tissues [13]. The substitution of larger Ca^2+^ ions with smaller Mg^2+^ ions induces lattice distortion, facilitating the phase transformation of HAp into the more soluble and bioactive β-tricalcium phosphate (β-TCP) phase during calcination and sintering at high temperatures [14]. Previous studies have shown that magnesium-doped HAp enhances interactions between implant materials and cells, promoting the formation of new tissues both in vitro and in vivo [15].

Sr^2+^ ions, found in the structure of bones and teeth, are closely linked to the bone’s response to osteoporosis. These ions promote the formation of new bone tissue while simultaneously reducing bone resorption, thus playing a vital role in maintaining bone density and strength [16]. Consequently, incorporating Sr^2+^ into the structure of HAp may influence certain physical and chemical properties of bioceramic materials [17]. Additionally, therapeutic effects of Sr^2+^ on bone-related conditions such as osteoporosis, osteoarthritis, and bone metastasis have been determined [18]. Materials doped with Sr^2+^ could potentially be utilized for local ion-delivery to improve treatment of the aforementioned conditions. Previous research has confirmed that co-doping with Sr^2+^ and Mg^2+^ leads to thermal stabilization of the β-TCP phase by suppressing its phase transition to α-tricalcium phosphate (α-TCP) when calcined at 1000 °C, thereby enhancing the mechanical properties of bioceramic materials [19].

F^−^ ions exist as an element in trace amounts in the mineral phase of bone and teeth, playing a crucial role in stimulating bone proliferation and differentiation [20]. F^−^ ions can replace OH^−^ groups [21], inducing increased crystallinity, organization of the apatite structure, and resistance to acidic dissolution [22]. It has been shown that replacing OH^−^ groups with F^−^ leads to enhanced protein adsorption and increased binding between cells and materials [23].

The hydrothermal method enables the production of HAp powder with a higher degree of crystallinity and well-defined, uniform particles with rod-like morphology mimicking natural apatite [14,19,24]. Scaffolds present macroporous structures that play a vital role in bone regeneration by acting as a cell support and allowing migration of cells and transport of nutrients and oxygen for proper vascularization, osteoconductivity, and the regulation of biological fluids, cells, and tissue influx into the implanted structure [25,26]. The sponge replica method, used for scaffold fabrication, is a simple, cost-effective technique that allows for the fabrication of highly porous structures with interconnected pores that resemble natural bone tissue [27].

While open interconnected porosity in macroporous ceramics is ideal for cell and tissue growth, it results in relatively poor mechanical properties, limiting their use to non-load-bearing applications [28]. In order to address this, scaffolds can be coated with polymers that can be biodegradable or stable in physiological conditions [29,30]. Chitosan, a natural linear polysaccharide derived from chitin, is recognized for its biodegradability, biocompatibility, and biological properties, including the promotion of cell proliferation and antibacterial activity. These characteristics make it a promising material for applications in bone tissue engineering and drug delivery systems [31,32].

Hydrazone compounds, particularly those containing heterocyclic moieties, have demonstrated significant anticancer properties, with cytotoxic effects observed against various tumor cells, including breast cancer, bone cancer, and myelogenous leukemia [33]. Furthermore, hydrazone-based anticancer drugs are emerging as a promising option when released from scaffolds, highlighting their potential in targeted cancer therapy [34]. In our previous work [35], we investigated a polymeric carrier based on poly(methacrylic acid) and casein for delivery of a dihydropyrimidinone-based compound for leukemia treatment. That investigation showed prolonged and controlled release of the dihydropyrimidinone derivative, with in vitro cytotoxic activity assays demonstrating great potential for leukemia therapy. Dihydropyrimidinones (DHPMs) are known to exert anticancer effects primarily by inhibiting human kinesin Eg5, causing mitotic arrest and apoptosis, with additional possible targets including centrin, calcium channels, and topoisomerase I [36]. Similarly, pyridone derivatives possess diverse pharmacological activities, including selective cytotoxicity against cancer cells, potentially due to their structural similarity to nucleic acid bases and their ability to interact with cellular targets involved in cancer progression [37]. Building on these findings, we have now expanded our research to investigate the synthesis and controlled release of a novel pyridone–dihydropyrimidinone-based hydrazone compound from a chitosan-coated calcium phosphate scaffold for localized antitumor therapy.

Building on prior advances in ion-doped HAp [38,39,40], polymer coatings [41], and tissue-engineering drug delivery systems [42,43], this study aims to introduce a novel hydrazone compound and incorporate it into a chitosan coating on a Mg-, Sr-, and F-doped HAp scaffold, establishing a dual-function platform that provides structural support for bone regeneration while enabling controlled, localized release of the anticancer drug for potential osteosarcoma therapy. In this study, a novel hydrazone drug was synthesized and characterized for its antitumor properties. Additionally, the influence of fluoride incorporation (0–2 mol.%) into HAp was investigated, and the most favorable composition was selected for scaffold fabrication, coating, and further characterization of mechanical properties, cytotoxicity, and drug release profile.

## 2. Materials and Methods

### 2.1. Mg-, Sr-, and F-Doped Hydroxyapatite Powder Synthesis

Hydroxyapatite doped with magnesium, strontium, and fluoride at different concentrations (Mg, Sr-HAp and Mg, Sr, xF-HAp, where x = 0.5, 1, or 2 mol%) was synthesized using a hydrothermal method reported elsewhere [44,45]. The chemical sources utilized in this process included calcium nitrate tetrahydrate (Ca(NO_3_)_2_·4H_2_O, Acros Organics, Geel, Belgium), sodium dihydrogen ethylenediaminetetraacetic acid dihydrate (Na_2_H_2_EDTA·2H_2_O, Acros Organics, Geel, Belgium), sodium dihydrogen phosphate dihydrate (NaH_2_PO_4_·2H_2_O, VWR International, Radnor, PA, USA), and urea (Acros Organics, Geel, Belgium). The salts magnesium nitrate hexahydrate (Mg(NO_3_)_2_·6H_2_O, CARLO ERBA Reagents, Cornaredo, Italy) and strontium nitrate (Sr(NO_3_)_2_, Acros Organics, Geel, Belgium), along with sodium fluoride (NaF, Riedelde Haën, Seelze, Germany), were used as a source of dopant ions. All samples contained 11.10 g Ca(NO_3_)_2_·4H_2_O, 11.80 g of EDTA, and 12.00 g of urea. The initial masses of Mg(NO_3_)_2_∙ 6H_2_O and Sr(NO_3_)_2_ were 0.385 g and 0.317 g, respectively. Table 1 presents the masses of NaH_2_PO_4_·2H_2_O and NaF salts used in the synthesis, which were varied in the precursor solutions relative to the nominal composition. The (Ca + Mg + Sr)/(P + F) molar ratio value was 1.67 in all samples. The precursor solutions were treated in an autoclave at 160 °C, under a pressure of 8 bar for 3 h. The obtained precipitates were collected by vacuum filtration, flushed with deionized water, and dried at 105 °C for 5 h. Mg^2+^ and Sr^2+^ ions were utilized for the partial substitution of Ca^2+^ ions in the hydroxyapatite structure. Concurrently, it was hypothesized that OH^−^ ions would be replaced by F^−^ ions throughout the hydrothermal synthesis process. The concentrations of Mg^2+^ and Sr^2+^ ions were maintained consistently at 3 mol.% each, relative to the concentration of Ca^2+^ ions across all doped samples. A previous study by Matic et al. [19] demonstrated that varying Sr/Mg ratios significantly affected the physicochemical properties of HAp and identified 3 mol% Sr and 3 mol% Mg as the optimal doping level. To investigate the effect of fluoride incorporation (within the range of 0–2 mol%) in this multi-ion-doped system, the Sr and Mg content was therefore kept constant during powder synthesis.

### 2.2. Processing of the Scaffolds

Mg, Sr, 1F-HAp powder was selected for scaffold processing, due to its optimal elemental and phase composition, and further calcined at 1000 °C for 2 h with a heating rate of 10 °C/min.

**Figure 1:**
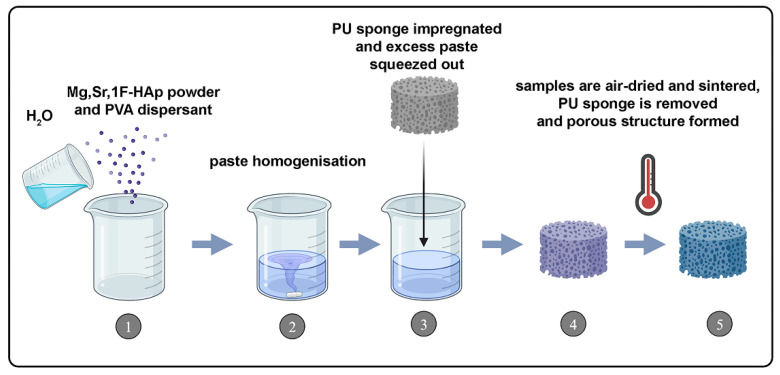
The scheme of sponge replica method. (1,2) Mg, Sr, F-HAp powder and PVA dispersant are mixed with water to form a homogeneous paste. (3) PU sponge is impregnated with the paste, and excess paste is removed to coat the sponge. (4,5) Samples are air-dried and sintered, removing the PU sponge to obtain the final porous scaffold.

Scaffolds were fabricated by the sponge replica method (Figure 1). The paste, consisting of 1.00 g of Mg, Sr, 1F-HAp powder, 0.10 g of polyvinyl alcohol, and 2.23 g of water, was treated in an ultrasonic bath, and then cylindrical macroporous polyurethane sponge cutouts (10 mm in radius) were submerged in the paste for even distribution. Excess paste in the sponge was squeezed out and left to dry at room temperature. The dried sponges were sintered in two steps to obtain scaffolds based on Mg, Sr, 1F-HAp. The heating process began with a rate of 2 °C/min until the temperature reached 750 °C, where it was held for 20 min to ensure complete burning of the sponge. Afterward, the heating rate was increased to 10 °C/min until reaching the final sintering step at 1400 °C, and the scaffolds were held for 2 h. The obtained scaffolds were coated with a thin layer of chitosan (Sigma-Aldrich, St. Louis, MO, USA, MW 50,000–190,000 Da, 75–85% deacetylated), prepared as 1% solution in acetic acid. The scaffolds were immersed in a preheated chitosan solution and left to soak for 2 h, ensuring that the temperature did not exceed 50 °C to prevent degradation of the polymer. The scaffolds were then taken out of the solution and air-dried at room temperature.

### 2.3. Synthesis of Hydrazone Drug Candidate

All chemicals used for the synthesis of novel hydrazone drug candidate were obtained from commercial sources and were of reagent-grade purity or better. The structure of obtained hydrazone was confirmed by ATR-FTIR and NMR analysis. Fourier-transform infrared (FTIR) spectra of the synthesized hydrazone were recorded in absorbance mode utilizing a Nicolet™ iS™ 10 FT-IR Spectrometer (Thermo Fisher Scientific, Waltham, MA, USA) with Smart iTR™ Attenuated Total Reflectance sampling accessories. The spectra were collected over a range of 500 to 4000 cm^−1^, with each spectrum consisting of 20 scans to enhance signal quality. ^1^H and ^13^C nuclear magnetic resonance (NMR) spectra were recorded using a Bruker Ascend 400 (400 MHz) spectrometer. Dimethyl sulfoxide-d_6_ was used as the solvent, and chemical shifts are reported in parts per million (δ).

**Figure 2:**
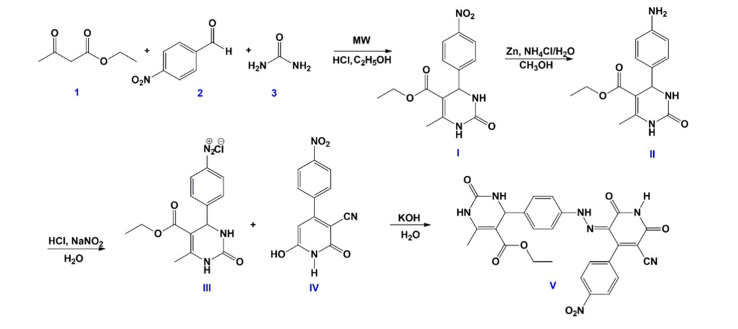
The synthetic scheme, including the formation of the DHPM derivative (I), formation of the amino derivative (II), and the diazo coupling reaction of diazonium chloride (III) and pyridone derivative (IV) yielding the hydrazone product (V).

The synthesis of novel hydrazone was performed via a diazo coupling reaction, using our previously reported method [33], following the synthetic pathway given in Figure 2. In detail, ethyl acetoacetate (1) (4 mmol, 0.510 mL), 4-nitrobenzaldehyde (2) (4 mmol, 0.604 g), and urea (3) (4 mmol, 0.240 g) were dissolved in 10 mL of ethanol, to which 4 drops of hydrochloric acid were added. This reaction mixture was heated in a microwave reactor (Anton Paar Monowave 300, Anton Paar GmbH, Graz, Austria) at 120 °C for 30 min. After completion, the mixture was cooled to 4 °C and stored at this temperature for 24 h. The dihydropyrimidinone (DHPM) product (I) was isolated by filtration, washed with ethanol, dried, and recrystallized from ethanol. Then, I (2 mmol, 0.670 g) was dissolved in 40 mL of methanol, followed by the addition of zinc (20 mmol, 1.438 g) in portions and an aqueous solution of ammonium chloride (4 mmol, 0.235 g in 4 mL of water). The mixture was stirred under the reflux for 5 h, and the reaction was followed by thin-layer chromatography (TLC). After completion, the mixture was filtered, and the filtrate was cooled to 4 °C for 24 h. The resulting product (II) was collected by filtration, washed with ethanol, dried, and recrystallized from ethanol. Further II (1 mmol, 0.275 g) was dissolved in water (60 mL) and concentrated hydrochloric acid (3 mL) and cooled to 0–5 °C. A solution of sodium nitrite (1.1 mmol, 0.0759 g) in cold water (4 mL) was added dropwise over 15 min. The mixture was stirred for 1 h to yield the diazonium chloride derivative (III). Pyridone (Py) compound (IV) was synthetized using previously reported method [46]. Obtained pyridone (1 mmol, 0.2572 g) was dissolved in an aqueous solution of potassium hydroxide (1 mmol, 0.056 g in 5 mL of water) and cooled to 0–5 °C. The diazonium chloride solution was added dropwise over 30 min to the pyridone solution with vigorous stirring. The reaction was stirred for an additional 3 h, maintaining the temperature between 0 and 5 °C. Upon completion, obtained hydrazone (V) was isolated by filtration, washed with water, dried, and recrystallized from ethanol, yielding an orange solid in 61%. ATR-FTIR (*ν*/cm^−1^): 3252 (NH), 2227 (CN), 1687 (CO), 1629 (CO), 1516 (NH); ^1^H NMR (400 MHz, DMSO-d_6_, *δ*/ppm): 14.61 (s, 1H, NH hydrazone), 12.29 (s, 1H, NH Py moiety), 9.18 (s, 1H, NH DHPM moiety), 8.42 (d, J = 8.7 Hz, 2H, Ar-H), 8.32 (d, J = 8.8 Hz, 2H, Ar-H), 8.16 (d, J = 8.8 Hz, 2H, Ar-H), 7.80 (d, J = 8.7 Hz, 2H, Ar-H), 7.69 (s, 1H, NH DHPM moiety), 5.09 (s, 1H, CH), 3.94 (q, J = 7.1 Hz, 2H, COOCH_2_CH_3_), 2.22 (s, 3H, CH_3_), 1.05 (t, J = 7.1 Hz, 3H, COOCH_2_CH_3_); ^13^C NMR (100 MHz, DMSO-d_6_, *δ*/ppm): 166.3 (COOCH_2_CH_3_), 165.6 (CO, Py moiety), 162.0 (CO, Py moiety), 161.1 (Py moiety), 159.3 (CO, DHPM moiety), 152.3 (C–CH3, DHPM moiety), 150.5 (Ar), 149.1 (Ar), 148.7 (Ar), 144.2 (Ar), 139.9 (Ar), 136.8 (Ar), 131.3 (Ar), 131.2 (Ar), 128.2 (Ar), 124.2 (Ar), 123.6 (C=N, Py moiety), 117.9 (Ar), 115.3 (C-CN), 99.2 (C-COOCH_2_CH_3_), 59.7 (COOCH_2_CH_3_), 54.0 (C–H), 18.3 (CH_3_, DHPM moiety), 14.5 (COOCH_2_CH_3_). The synthesized hydrazone compound has a molecular weight of 543.49 g·mol^−1^, which is relevant for calculating loading capacity in the scaffolds and for interpreting NMR signal intensities.

### 2.4. Antitumor Analysis of Hydrazone Compound

#### 2.4.1. Cell Culture

Rat osteosarcoma UMR106 cells (CRL-1661, ATCC, Manassas, VA, USA) and murine mammary carcinoma 4T1 cells (CRL-2539, ATCC, Manassas, VA, USA) were cultured at 37 °C in a humidified 5% CO_2_ atmosphere. Cells were maintained in Dulbecco’s Modified Eagle Medium (DMEM, Sigma Aldrich, Merck, St. Louis, MO, USA) supplemented with 10% (*v*/*v*) fetal bovine serum (FBS, Sigma Aldrich), 1% (*v*/*v*) penicillin/streptomycin solution (100 U/mL penicillin and 100 µg/mL streptomycin), and 2 mM L-glutamine (Sigma Aldrich).

#### 2.4.2. Preparation of Compound for Antitumor Test

Hydrazone compound was dissolved in dimethyl sulfoxide (DMSO, Sigma Aldrich) to obtain a 40 mM stock solution. Working solutions were freshly prepared by diluting the stock with complete culture medium to the desired concentrations (200–1.5625 µM). The final concentration of DMSO in culture medium did not exceed 1% (*v*/*v*). Cisplatin (CDDP, Sigma Aldrich) was dissolved in sterile phosphate-buffered saline (PBS) to prepare a 10 mM stock solution, which was further diluted with complete medium for treatments.

#### 2.4.3. Antitumor Test

The cytotoxic effect of hydrazone compound was assessed by the MTT assay according to [47] with slight modifications. Briefly, UMR106 and 4T1 cells were seeded in 96-well plates at a density of 5 × 104 cells per well in 100 µL of complete medium and allowed to adhere overnight. Cells were then exposed for 24 h to increasing concentrations of hydrazone compound (200–1.5625 µM). Cisplatin (CDDP) was used as the reference drug, and untreated cells served as negative controls.

Following incubation, 10 µL of 15% MTT solution (5 mg/mL in PBS; Sigma Aldrich) was added to each well, and the plates were incubated for 4 h at 37 °C. The supernatant was carefully removed, and 150 µL of dimethyl sulfoxide (DMSO, Sigma Aldrich) was added to solubilize the formazan crystals. The plates were shaken for 10 min at room temperature, and absorbance was measured at 595 nm using a microplate Zenyth 3100 Multimode detector. Cell viability was expressed as a percentage relative to untreated controls after subtraction of background values using the following formula:

% cytotoxicity = 100 − ((E  −  B)/(S  −  B) × 100),

where B represents the background of the medium alone, S denotes the total viability/spontaneous death of untreated target cells, and E indicates the experimental well. The experiments were performed in triplicate, according to ISO 10993-5 (2009) [48] recommendations. Nonlinear regression of log-transformed concentration data was used to calculate IC_50_ values.

### 2.5. Incorporation of Hydrazone into Chitosan Coating and Scaffold Treatment

A 2% (*w*/*v*) chitosan solution was prepared by dissolving 0.5 g of chitosan in 25 mL of 1% (*v*/*v*) acetic acid. The solution was stirred on a magnetic stirrer at 60 °C until a clear and homogeneous solution was obtained. Subsequently, 20 mg of hydrazone was added to the chitosan solution, and stirring was continued at 60 °C to ensure uniform dispersion of the drug.

The resulting chitosan–hydrazone blend was used to coat the previously printed and dried scaffolds. Scaffolds were fully immersed in the prepared solution and incubated at room temperature for 3 h without agitation. After immersion, scaffolds were removed from the solution, placed on a sterile surface, and air-dried at room temperature until complete solvent evaporation.

### 2.6. Characterization of Sample Powders

#### 2.6.1. Energy-Dispersive X-Ray (EDX) Analysis

Energy-dispersive X-ray (EDX) analysis was used to determine the elemental composition of the powders and to confirm incorporation of Mg^2+^, Sr^2+^, and F^−^ ions in the powder samples. The analysis was performed on an INCAPentaFETx-3 device coupled with SEM Tescan Vega TS 5130MM (Tescan s.r.o., Brno, Czech Republic), operated at voltage of 20 keV. All obtained results are presented as the average arithmetic value of three measurements and expressed in atomic percentages. The used magnification was 1000 times.

#### 2.6.2. X-Ray Diffraction (XRD) Analysis

X-ray diffraction (XRD) analysis was used for phase identification of samples before and after calcination. The analysis was performed on an X-ray diffractometer (Rigaku Smartlab), where the angle 2θ was ranged from 20° to 70°, with a scan step of 0.02° s^−1^. The obtained XRD patterns were compared with standards compiled by the Joint Committee on Powder Diffraction Standards cards, JCPDS 09–0169, JCPDS 09–0348, and JCPDS 09–0432 for β−TCP, α−TCP, and HAp, respectively, in order to perform crystallographic identification, while Profex 5.4.1. software was used for phase quantification.

#### 2.6.3. Scanning Electron Microscopy (SEM)

A scanning electron microscope Tescan FE-SEM Mira 3 KSMU (Tescan s.r.o., Brno, Czech Republic), operated at 20 keV, was used to examine the morphology of nanostructured powders. Before SEM analysis, all samples were coated with a thin gold layer using a sputter coater (Polaron SC503, Fisons Instruments, Ipswich, UK).

### 2.7. Characterization of Scaffold Samples

#### 2.7.1. Structural Analysis of Mg, Sr, 1F-HAp Scaffolds

The phase compositions of the Mg, Sr, 1F-HAp scaffolds after sintering at 1400 °C were examined using X-ray diffraction (XRD). The measurements were carried out with a Rigaku Smartlab diffractometer, within the 2θ range of 20° to 70°, applying a scan step of 0.02° s^−1^. The obtained diffraction patterns were interpreted by comparison with reference data from the Joint Committee on Powder Diffraction Standards (JCPDS) cards: 09–0169 for β-TCP, 09–0348 for α-TCP, and 09–0432 for HAp. Quantitative phase analysis was performed using Profex software version 5.4.1.

#### 2.7.2. Microstructures of Uncoated and Coated Mg, Sr, 1F-HAp Scaffolds

Microstructures of uncoated and polymer coated scaffolds were determined by using FE-SEM, applying the same scanning conditions as for the HAp powders. ImageJ 1.54g software was used to measure the pore sizes from SEM images of both uncoated and coated scaffolds, determining both the minimum and maximum diameters based on measurements of 20 pores per sample (n = 20).

#### 2.7.3. Mechanical Properties of Mg, Sr, 1F-HAp Scaffolds (Uncoated and Coated with Chitosan)

The compressive strength of both uncoated and chitosan-coated scaffolds was evaluated in the dry state using a Universal Testing Machine AG-X Plus (Shimadzu, Kyoto, Japan), equipped with a load cell with a capacity of up to 1000 N (force range: 0.01–1000 N). Contact between the testing tool and the sample was automatically detected by applying a predefined contact force of 0.02 N. Compression tests were performed at a crosshead speed of 10 mm/min at room temperature. The compressive strength (σc) was determined from the standard stress–strain curves at the fracture point, up to 20% deformation. The results are reported as the arithmetic mean of three independent measurements.

#### 2.7.4. Fourier-Transform Infrared Spectroscopy (FTIR) of Uncoated Mg, Sr, 1F-HAp Scaffolds, Chitosan, and Chitosan-Coated Mg, Sr, 1F-HAp Scaffolds

FTIR analysis was performed to compare the spectra of uncoated Mg, Sr, 1F-HAp scaffolds, pure chitosan, and Mg, Sr, 1F-HAp scaffolds coated with chitosan. The measurements were carried out using a Nicolet™ iS™10 FTIR spectrophotometer (Thermo Fisher Scientific, Waltham, MA, USA) with the attenuated total reflectance (ATR) method, in the wavenumber range from 400 to 4000 cm^−1^, applying a resolution of 4 cm^−1^ and 20 scans per sample. Data acquisition and processing were performed using the Omnic 9 software package. Chitosan was analyzed in its powder form, while the scaffolds were dried at room temperature and finely ground before recording the spectra.

#### 2.7.5. In Vitro Bioactivity of Scaffolds

In vitro bioactivity represents the ability of the material to induce formation a new layer of HAp crystals on the material surface when immersed in biological fluid. Bioactivity of uncoated and coated scaffolds was examined by incubation of scaffolds in a Kokubo simulated body fluid (SBF) [49]. The samples of scaffolds immersed in SBF were incubated in a period of 28 days at a temperature of 37 °C. The SBF was changed every three days. Formation of a new layer of hydroxyapatite on the scaffolds‘ surface after incubation in SBF analyzed by Tescan FE-SEM Mira 3 KSMU (Tescan s.r.o., Brno, Czech Republic), applying the same scanning conditions as previously mentioned.

#### 2.7.6. In Vitro Cytotoxicity of the Coated Mg, Sr, 1F-HAp Scaffolds

In vitro cytotoxicity of the coated scaffolds was determined against normal human MRC-5 cells using MTT cell survival assay. Coated scaffolds were settled in the wells of a sterile 6-well plate for adherent cell cultures. The samples in the plate were sterilized under UV radiation in the chamber for 1 h. Human normal fetal lung fibroblasts MRC-5 were seeded in 5 mL of RPMI 1640 cell culture medium with 10% fetal bovine serum, containing L-glutamine (2 mM), streptomycin (100 μg/mL), penicillin (100 U/mL), and 4-(2-hydroxyethyl)-1-piperazineethanesulfonic acid (HEPES) (25 mM). Overall, 100,000 cells were seeded in each well. To the blank wells, only cell culture medium in an equivalent volume was added. The cell control sample contained only cells in culture medium. For this test, four wells were used, one of which was blank, while cells were added to the other three. The cell plates were then transferred to an incubator, and the cells were grown in the incubator at a temperature of 37 °C, in an atmosphere of humidified air with 5% SO_2_ for the next 72 h.

Survival of target cells was determined by MTT cytotoxicity assay after 72 h of cell incubation [50,51]. To each of the 4 wells, we added 333 µL of MTT reagent (5 mg/mL, dissolved in PBS), and then the samples were incubated for 4 h at 37 °C in a CO_2_ incubator. After incubation, 3333 µL of 10% SDS was added to the samples. The plates were then incubated at 37 °C in a CO_2_ incubator, and after 24 h, the samples were transferred to 96-well plates, and the absorbance was measured at a wavelength of 570 nm on a spectrophotometer, Thermo Scientific^TM^ Multiskan SkyHigh (Thermo Fisher Scientific, Waltham, MA, USA). Cell survival (%) was determined by dividing the absorbance (Au) of a sample of cells that grew in the presence of the scaffolds by the absorbance (Ak) of a control sample of cells that grew in the nutrient medium and multiplying by 100. During calculation, the read absorbance values of the blank samples were subtracted from the read absorbance values of the samples with cells. Cell survival was determined based on the formula S [%] = (Au/Ak)×100.

The MRC-5 cell line was obtained from the American Type Culture Collection (ATCC) (Manassas, VA, USA). The reagents used to maintain cell cultures were as follows: RPMI 1640, PBS, L-glutamine, HEPES, antibiotics, MTT, and SDS were the product of Sigma Aldrich (St. Louis, MO, USA). Thermo Scientific™ Biolite™ 6-well cell culture plates were used in the experiments. Data were obtained from three independent measurements and are presented as mean ± standard deviation.

#### 2.7.7. Hydrazone Compound

To test the scaffolds’ drug delivery properties, chitosan–hydrazone bland was prepared, and scaffolds were immersed to form a chitosan–hydrazone coating. Thereafter, release of hydrazone compound, from coated scaffolds, was measured by UV-Vis spectroscopy over 15 days. The release of hydrazone was analyzed by immersing the scaffolds in phosphate-buffered solution (PBS, pH = 7.4) at 37 °C to mimic human body conditions. The amount of hydrazone released in the PBS was measured in regular intervals over 15 days. Absorbance measurements of the released hydrazone in the medium were taken at 400 nm using a Shimadzu 1800 UV-Vis spectrophotometer (Shimadzu Corporation, Kyoto, Japan). The concentration (ppm) of the released hydrazone in a certain time of measurement was obtained by multiplying the obtained absorbance (at 400 nm) with the slope of the standard curve made for certain concentrations of the drug.

The release kinetics of drugs from the composites were evaluated using several mathematical models, including the zero-order model (*m_t_ = k*_0_ × *t*), first-order model (ln(1 − α) = −*k*_1_ × *t*), the Higuchi model (*m_t_* = *k_H_* × *t*^1/2^), and the Korsmeyer–Peppas model (α = *k_p_* × *t^n^*). In these equations, *m_t_* denotes the mass of the drug released at time *t*; α denotes the fraction of drug released at time *t*; *k*_0_ is the zero-order release constant; *k*_1_ is the first-order release constant; *k_H_* is the Higuchi dissolution constant; *k_p_* is the Korsmeyer–Peppas rate constant; and *n* is the diffusional exponent, which indicates the underlying drug release mechanism [52,53,54]

## 3. Results and Discussion

### 3.1. EDX Analysis of Mg, Sr, F-HAp Powders

EDX analysis was performed to investigate the elemental composition of the synthesized powders and confirm the presence of doped ions (Table 2). The primary components of the samples included oxygen, phosphorus, and calcium, alongside detectable amounts of magnesium, strontium, and fluoride ions. The analysis confirmed that all synthesized powders contained Mg^2+^ and Sr^2+^ dopants. Atomic percentages (at.%) for hydrothermally synthesized HAp powders, nominally doped with 3 mol.% of both Sr^2+^ and Mg^2+^ ions at a pressure of 6 bar, have been previously reported [19]. The reported composition included Ca at 17.11%, P at 14.11%, Mg at 0.30%, and Sr at 1.33%, with a Ca/P ratio of 1.21. In comparison, synthesis of Mg, Sr-HAp with the same nominal composition in this study was performed at higher pressure (8 bar), which may have influenced ionic incorporation and availability, leading to a lower Ca/P ratio and a reduced amount of incorporated Sr^2+^ ions, while Mg^2+^ content remained comparable. Furthermore, the presence of F^−^ ions was noted in Sr, Mg, 1F-HAp and Sr, Mg, and 2F-HAp samples with atomic percentages of 0.24%, and 1.34%, respectively. The Ca/P molar ratio in all samples was lower (1.16–1.18) than the stoichiometric ratio of HAp (1.67), indicating calcium-deficient HAp structure, which is consistent with previous findings [19], with slight variations attributed to the assumption that F^−^ ions replace OH^−^ ions rather than PO_4_^3−^ ions [55].

### 3.2. XRD Analysis of Mg, Sr, F-HAp Powders

**Figure 3:**
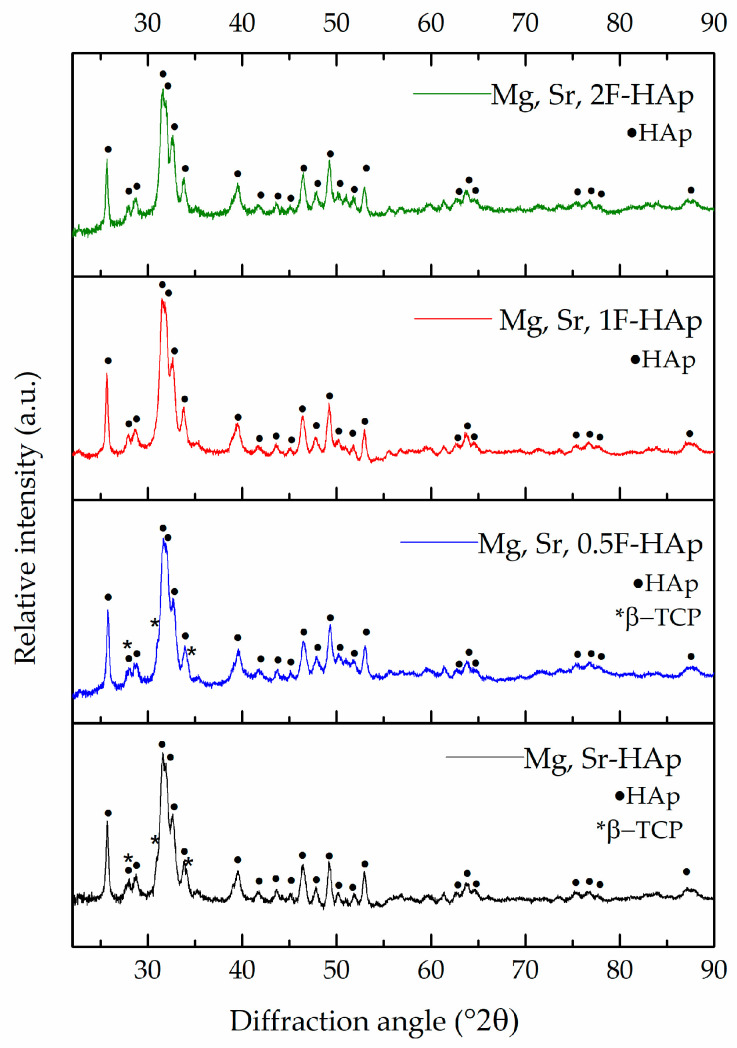
XRD patterns of Mg, Sr-HAp, Mg, Sr, 0.5F-HAp, Mg, Sr, 1F-HAp, and Mg, Sr, 2F-HAp nanopowders presented from bottom to top.

The results of XRD analysis of Mg, Sr-HAp and Mg, Sr, F-HAp powders provide critical insights into the effects of ion-doping on the phase composition of HAp powders, as revealed in the obtained X-ray diffractograms (Figure 3) and subsequent quantification (Table 3). As illustrated in Figure 2, the diffractograms of Mg, Sr-HAp and Mg, Sr, 0.5F-HAp powders not only display the dominant peaks of the HAp phase but also the peaks characteristic of the β-TCP phase. Further quantitative phase analysis confirmed the biphasic composition of the powders, with the HAp phase emerging as the predominant phase. This observation is consistent with findings from previous research [19], which established that the co-doping with Mg^2+^ and Sr^2+^ ions enhance the stability of the β-TCP phase.

On the other hand, the Mg, Sr, 1F-HAp and Mg, Sr, 2F-HAp samples were found to be monophasic hydroxyapatite, with the XRD diffractograms displaying peaks exclusively attributed to the HAp phase. The noteworthy stability of the HAp phase in these compositions can be primarily attributed to the influence of doping with F^−^ ions, which play a crucial role in stabilizing the structural integrity of HAp [56]. It can be attributed to the substitution of OH^−^ groups by F^−^ in the apatite lattice. F^−^ doping influenced a slight reduction in the a-axis lattice parameter of HAp, while the c parameter remained unchanged (see Supplementary Materials). This is in accordance with literature data on the F^−^ effect on HAp lattice parameters [57].

### 3.3. SEM Analysis of Mg, Sr, F-HAp Powders and Calcined Powder

**Figure 4:**
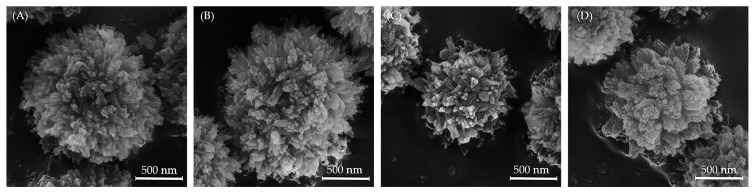
SEM micrographs of powder samples: (**A**) Mg, Sr-HAp, (**B**) Mg, Sr, 0.5F-HAp, (**C**) Mg, Sr, 1F-HAp, and (**D**) Mg, Sr, 2F-HAp.

SEM micrographs of the synthesized powders are shown in Figure 4 and show that all powders are composed of rod-like sub-particles agglomerated in spherical particles ranging from 500 to 1000 nm in size. Increasing the F^−^ ion content had no significant influence on the morphology of rod-like sub-particles and only led to a slight reduction in agglomerated spherical particle size. This morphological feature is consistent with the minimal changes observed along the a-axis, but not along the c-axis (see Supplementary Materials).

**Figure 5:**
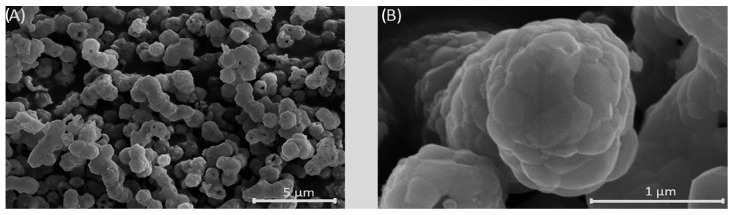
SEM micrograph of Mg, Sr, 1F-HAp calcined powder. (**A**) Magnification 10 kx; (**B**) magnification 80 kx.

Based on the elemental and phasic composition, for further scaffold processing, the Mg, Sr, 1F-HAp powder was selected. As seen in Figure 5, the calcination of the Mg, Sr, 1F-HAp powder resulted in slightly increased particle size due to grain formation and particle coalescence during the temperature treatment. The formation of interconnected particles can also be seen in previously reported studies [58].

### 3.4. XRD Analysis of the Mg, Sr, 1F-HAp Scaffolds

**Figure 6:**
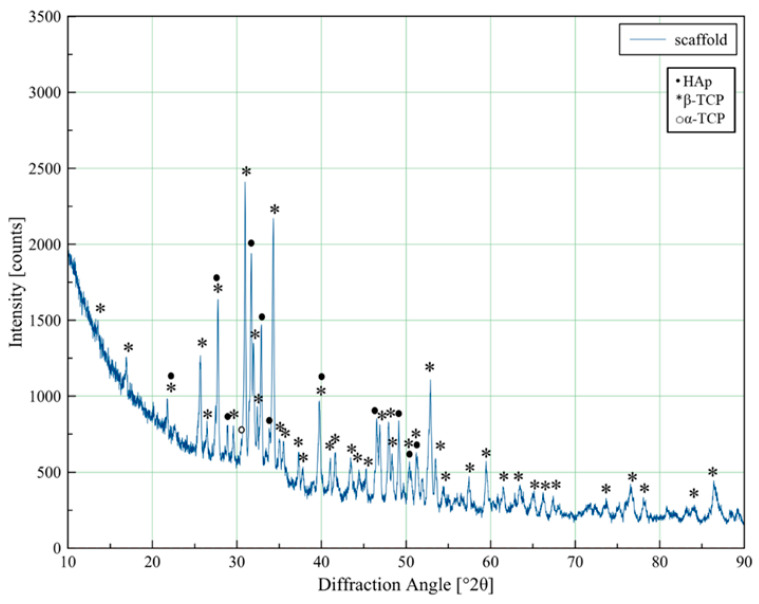
XRD diffractogram of Mg, Sr, 1F-HAp scaffold sintered at 1400 °C.

The identification and quantification of phases of sintered scaffold based on Mg, Sr, 1F-HAp powder was performed based on the XRD diffractogram of the scaffold sintered at 1400 °C (Figure 6). The quantification results provided a representation of phase proportions in weight percentages (wt.%), as illustrated in Table 4, indicating the dominant phase was β-TCp (62.73%), followed by HAp (33.14, along with small amounts of α-TCP (4.13%). The stabilization of the β-TCp phase highlights the significant influence of doping with Mg^2+^ and Sr^2+^ ions. By carefully optimizing the concentrations of Mg^2+^ and Sr^2+^ ions, it is feasible to prevent the formation of the α-TCP phase, which often emerges during the processing of materials at elevated temperatures, thus ensuring the stability of the desired β-TCp phase [19]. These findings are essential for enhancing bioactivity and bone regeneration, considering that the β-TCp phase exhibits superior solubility and bioactivity compared to the HAp phase [59,60].

### 3.5. Structure of the Uncoated and Coated Mg, Sr, 1F-HAp Scaffolds

**Figure 7:**
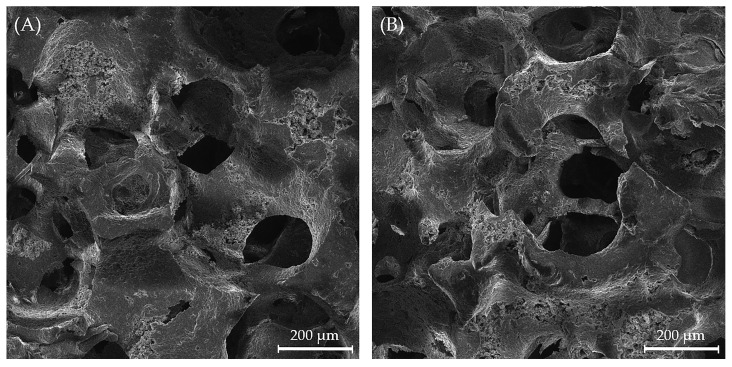
SEM micrographs of (**A**) uncoated Mg, Sr, 1F-HAp scaffold and (**B**) Mg, Sr, 1F-HAp scaffold coated with chitosan.

Figure 7 shows the uniform macroporous structure of the obtained scaffolds. It can be observed that the chitosan coating did not disrupt the macroporous structure with interconnected macropores. For uncoated scaffolds, the pore sizes ranged from 45 to 306 μm, with an average pore size of 122 ± 51 µm in diameter, and for coated scaffolds, the pore sizes ranged from 47 to 227 μm, with an average pore size 125 ± 53 µm in diameter, which makes them promising cell carrier candidates with adequate porosity for cell growth and proliferation [27].

The scaffold microstructure and pore size are crucial parameters influencing in vivo and in vitro performance. The obtained pore sizes fall within the range considered optimal for bone tissue regeneration. Smaller pores (~45–100 μm) could support cell adhesion and proliferation, while larger pores (~100–300 μm) facilitate vascularization and tissue ingrowth. Previously, it was demonstrated that the morphological analysis of the scaffolds highlighted their porous characteristics, which are ideal for drug retention and provide a high specific surface area conducive to interactions with both cells and drugs [61]. Additionally, the incorporation of biodegradable polymers, such as chitosan, as a coating on the scaffolds significantly enhanced the drug release properties by facilitating a prolonged release duration, ultimately improving the therapeutic effectiveness.

**Figure 8:**
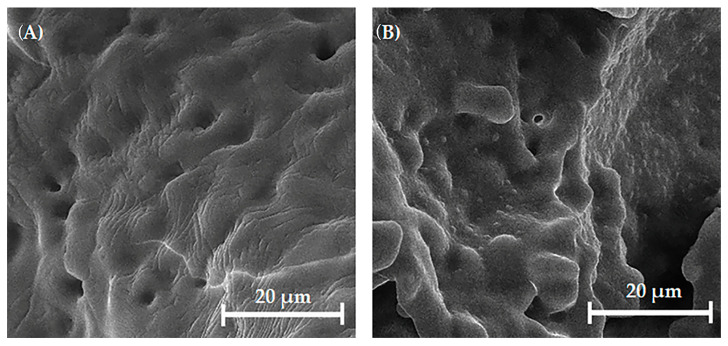
SEM micrographs of the surface of (**A**) uncoated Mg, Sr, 1F-HAp scaffold and (**B**) Mg, Sr, 1F-HAp scaffold coated with chitosan.

In Figure 8A, the SEM micrograph shows the surface of the uncoated HAp scaffold, which appears relatively smooth with visible micropores. In contrast, Figure 8B presents the SEM micrograph of the chitosan-coated HAp scaffold, revealing a rougher and more irregular surface morphology with distinguishable globular or granular features—presumably corresponding to deposited chitosan. These structures are distributed across the surface and partially fill the micropores, likely contributing to reduced porosity. The chitosan coating appears relatively uniform, forming a continuous layer.

**Figure 9:**
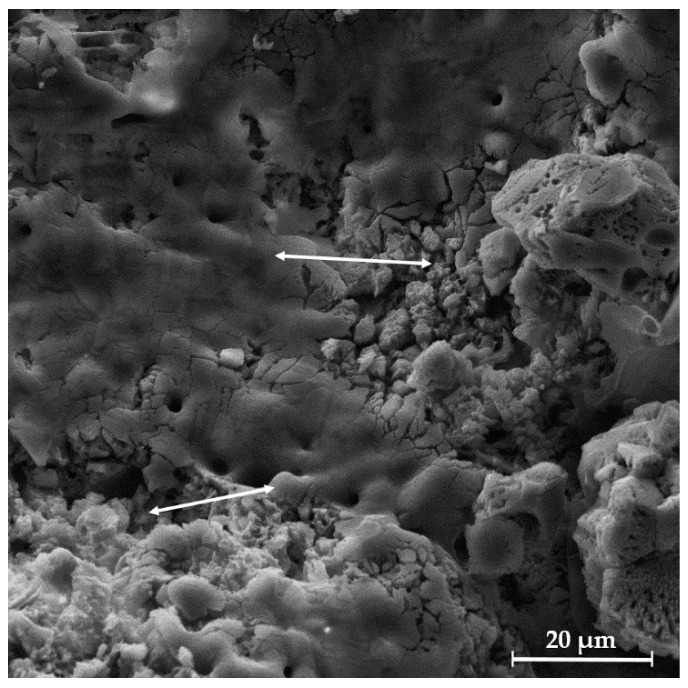
SEM micrograph of chitosan-coated Mg, Sr, 1F-HAp scaffold fracture revealing coated and uncoated regions.

These observations are further supported by the fracture surface morphology of the coated scaffold (Figure 9), where a noticeable difference in the surface appearance is visible in the region where the coating was delaminated (indicated by arrows) compared to coated regions. The observed reduction in microcracking and finer surface texture of the coated scaffolds suggests the formation of a stable and continuous polymeric layer. The improved coating uniformity is likely a result of physical interactions between chitosan and HAp, possibly through hydrogen bonding between the amino groups of chitosan and the hydroxyl groups on the HAp surface [30,62].

### 3.6. FTIR Analysis of Uncoated Mg, Sr, 1F-HAp Scaffolds, Chitosan, and Chitosan-Coated Mg, Sr, 1F-HAp Scaffolds

**Figure 10:**
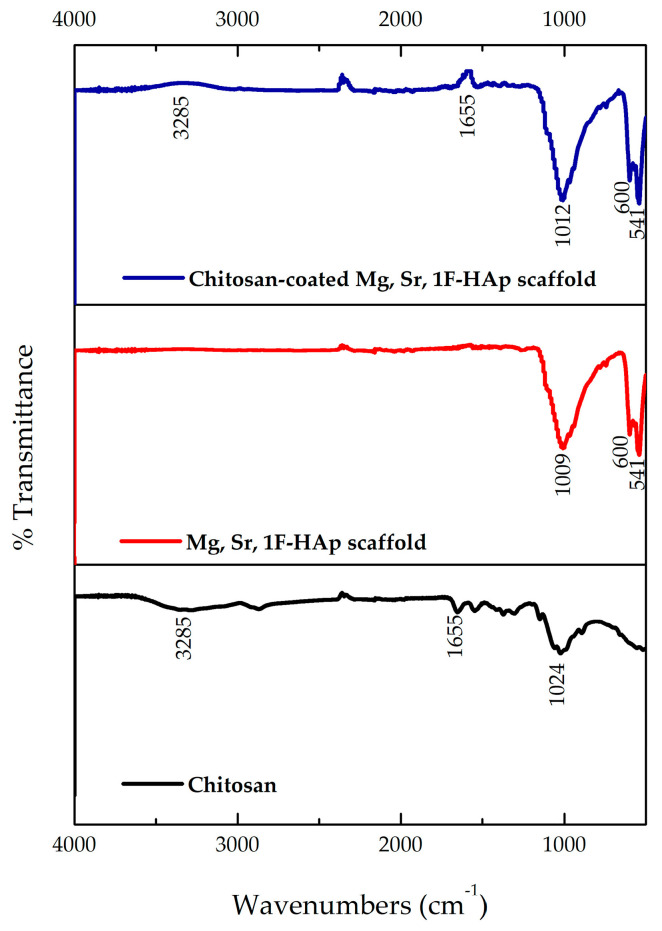
FTIR spectra of uncoated Mg, Sr, 1F-Hap scaffold, pure chitosan, and chitosan-coated Mg, Sr, 1F-Hap scaffold.

Figure 10 presents a comparative overview of the FTIR spectra of uncoated Mg, Sr, 1F-HAp scaffolds, pure chitosan, and chitosan-coated Mg, Sr, 1F-HAp scaffolds.

FTIR analysis revealed that pure HAp exhibited characteristic peaks at 1008.5 cm^−1^, 599.81 cm^−1^, and 541.15 cm^−1^, corresponding to phosphate (P–O) vibrations typical of the apatite structure [62]. These peaks persisted following chitosan coating, albeit slightly shifting to 1011.59 cm^−1^, 599.94 cm^−1^, and 541.86 cm^−1^. This confirms that the HAp crystalline structure was preserved. The shift and enhancement, in peak 1011.59 cm^−1^, can be attributed to the contribution of chitosan’s C–O–C stretching vibration (originally at 1023.71 cm^−1^) [63], indicating a strong interaction between chitosan glycosidic bonds and the HAp surface.

Additionally, the chitosan spectrum displayed typical peaks at 1545 cm^−1^ (Amide II, NH_2_ groups) and 1654 cm^−1^ (Amide I, C=O, and NH_2_ deformation) [63]. These peaks shifted slightly and lost intensity in the chitosan-coated HAp samples. The Amide II peak moved from 1545.96 cm^−1^ to 1528.55 cm^−1^, suggesting that amino groups were involved in hydrogen bonding with HAp.

The overlapping -OH and -NH_2_ stretching vibrations in chitosan are responsible for the broad band at 3284 cm^−1^, which also showed changes in shape and intensity after coating. This further supports the formation of hydrogen bonds and validates the physical interactions between chitosan and the HAp surface.

### 3.7. Mechanical Properties of Uncoated and Coated Mg, Sr, 1F-HAp Scaffolds

**Figure 11:**
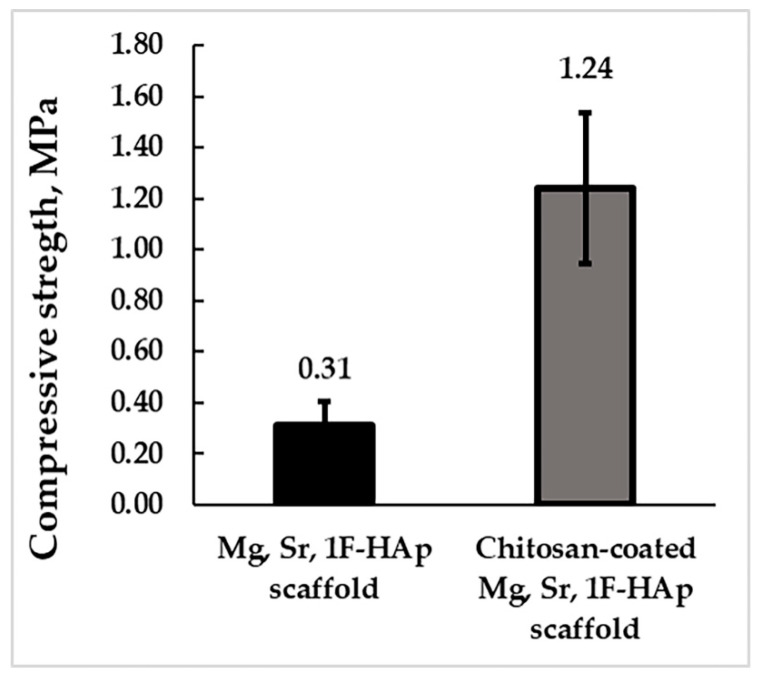
Compressive strengths of the Mg, Sr, 1F-HAp scaffolds (uncoated and chitosan-coated).

As shown in Figure 11, the Mg, Sr, 1F-HAp scaffolds had an average compressive strength of 0.31 ± 0.09 MPa, whereas the chitosan polymer coating improved the compressive strength, increasing it to 1.24 ± 0.30 MPa. The compressive strength of HAp scaffold is lower [64] due to the inherent brittleness of hydroxyapatite scaffolds [65]. The HAp scaffolds coated with chitosan exhibited significantly improved mechanical properties due to the flexible polymer coating, which reduced the potential for crack growth and enhanced the scaffold’s deformability. The chitosan-based coatings acted as a reinforcing layer by partially filling the pore walls and bridging microcracks, as observed in SEM images (Figure 8), thus improving the mechanical integrity. The excellent wettability and compatibility between HAp and chitosan resulted from interactions between the amino and hydroxyl groups of chitosan and the hydroxyl groups and calcium ions on the surface of the HAp. These interactions contributed to notable improvements in both the mechanical strength and biocompatibility of the scaffolds [66]. Comparable results have been reported, showing a compressive strength of 0.207 MPa for Mg- and Sr-doped hydroxyapatite scaffolds coated with gelatin [67]. Previous studies have shown that HAp/chitosan scaffolds exhibited a compressive strength of 0.63 ± 0.07 MPa at 15% strain, highlighting the significant influence of chitosan in enhancing the mechanical properties of the scaffolds [68]. Although the compressive strength of the coated scaffolds is not sufficient for load-bearing applications, these results highlight the positive effect of the polymer coating on scaffold mechanical stability.

### 3.8. In Vitro Bioactivity of Uncoated and Coated Scaffolds

**Figure 12:**
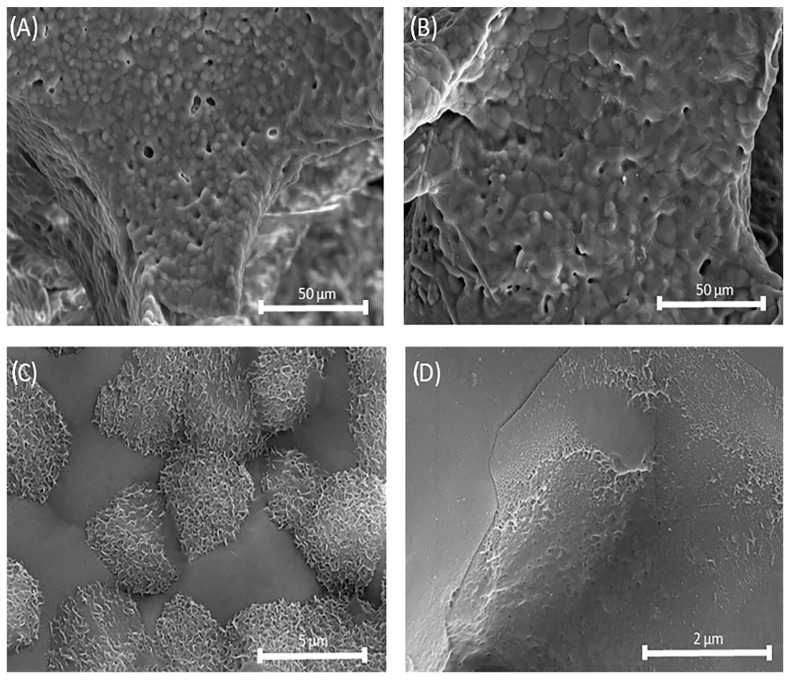
SEM micrographs of Mg, Sr, 1F-HAp scaffolds after bioactivity testing: (**A**,**C**) without chitosan coating; (**B**,**D**) with chitosan coating.

After immersion of Mg, Sr, 1F-HAp scaffolds for 28 days in SBF at 37 °C under stationary conditions, scaffolds without the chitosan coating showed good bioactivity. As seen on the SEM micrograph (Figure 12C), hydroxyapatite crystals formed on the surface of the Mg, Sr, 1F-HAp scaffold, confirming adequate bioactivity which is beneficial for potential application in bone tissue engineering. Mg and Sr can induce changes in the crystal structure, including decreased crystallinity and increased solubility, which may affect the ion release rate and the bioactivity of the material [69,70,71]. In contrast, fluoride substitution stabilizes the apatite structure, enhancing thermal stability and reducing solubility, thereby potentially improving the formation of a hydroxyapatite layer on the material surface [72]. On the other hand, a lower amount of newly formed HAp crystals was found on the surface of the chitosan-coated Mg, Sr, 1F-HAp scaffolds (Figure 12D). Delayed in vitro bioactivity of the coated scaffolds was likely caused by the slow degradation of non-bioactive chitosan in the SBF under static conditions. To achieve optimal mechanical and bioactive properties in HAp-based scaffolds, the imperative is to elucidate the degradation kinetics of the polymer coating. This understanding is critical for developing an effective polymer-coating strategy utilizing polymers with suitable degradation rates. Furthermore, it is noteworthy that the dissolution of these polymers under physiological in vivo conditions can transpire at significantly accelerated rates due to the action of various enzymatic factors [73].

### 3.9. In Vitro Cytotoxicity of Coated Mg, Sr, 1F-HAp Scaffolds

To ensure the lack of toxic effects of the fabricated scaffolds coated with chitosan, the in vitro cytotoxicity toward normal lung fibroblasts MRC-5 was tested by the MTT assay. It was found that these scaffolds had not negatively affected cell survival. On the contrary, the mean cell survival of MRC-5 cells in the presence of the Mg, Sr, 1F-HAp scaffolds coated with chitosan was increased up to 106.33 ± 6.58% (Table 5) compared to the control cell sample (100%).

A previous study reported that C2C12 cells showed significant reductions in viability when exposed to the scaffold elute medium, with viability ranging from 31.6% to 76.4%, depending on the composition of the chitosan/HAp/FAp scaffolds [74]. In contrast, the MgSrF-HAp scaffolds obtained in this study coated with chitosan did not exert cytotoxicity on normal MRC-5 cells.

### 3.10. Structural Characterization of Hydrazone Compound

The ATR-FTIR spectra confirmed that the synthetized molecule adopts the hydrazone form in the solid state. Stretching vibrations of the carbonyl groups are observed in the region of 1687–1629 cm^–1^, while the N–H stretching vibration of hydrazone bond appear at 3252 cm^–1^. Stretching vibrations of –C≡N group appear at 2227 cm^–1^. The band at 1516 cm^–1^ is ascribed to mutual vibrations of N–H group bending and C=N stretching, which further support the presence of hydrazone form in solid state. The structure of hydrazone in DMSO-d_6_ was confirmed by recording the ^1^H and ^13^C NMR spectra. Analysis of the ^1^H NMR spectrum verified the presence of all expected protons. The signal of the hydrazone N–H group is observed at 14.61 ppm. The ^13^C NMR spectrum also corresponded to the predicted structure and confirmed the presence of hydrazone form, evidenced by signals of two carbonyl carbons, originating from the pyridone moiety at 165.6 ppm and 162.0 ppm (Figure S1). This confirmed compound stability, the compound is present in the solid state (ATR-FTIR spectroscopy), as well as in solution (NMR spectroscopy), in hydrazone form, which represents the most stable tautomer of the synthesized molecule [33,75,76].

### 3.11. Antitumor Assessment of Hydrazone Compound

**Figure 13:**
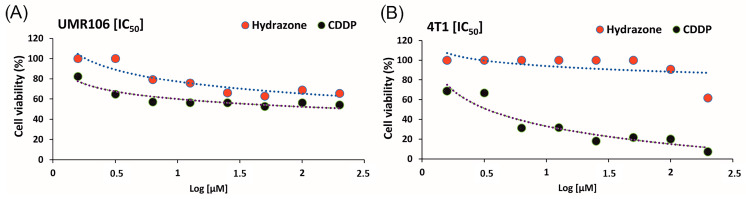
Dose–response curves of hydrazone and cisplatin (CDDP) in UMR106 osteosarcoma cells (**A**) and 4T1 breast cancer cells (**B**). Cell viability was assessed by MTT assay after 24 h of treatment with increasing concentrations of compounds (1.5625–200 µM). Data are expressed as mean percentage values, shown with nonlinear regression fits used for IC_50_ estimation.

The MTT assay demonstrated that both hydrazone and cisplatin (CDDP) reduced the viability of UMR106 osteosarcoma cells after 24 h of exposure (Figure 13A). Hydrazone compound decreased cell viability from 75.35% at lower concentrations to 58.76% at higher concentrations, while cisplatin reduced viability from 65.81% to 55.79%. No statistically significant difference in cytotoxicity was observed between hydrazone and CDDP at higher concentrations (*p* > 0.05). Regression analysis confirmed a statistically significant dose-dependent reduction in viability for both compounds, with a stronger effect observed for L2 (R^2^ = 0.258, B = –2.321, *p* < 0.001) compared to cisplatin (R^2^ = 0.158, B = –1.630, *p* = 0.001).

In 4T1 breast cancer cells, hydrazone compound also exerted a cytotoxic effect (Figure 13B). Cell viability decreased markedly at higher concentrations, showing cell viability of 47.84% at the highest tested concentration. Regression analysis confirmed a strong and statistically significant dose-dependent effect (R^2^ = 0.584, B = –14.496, *p* < 0.001). Cisplatin also displayed a robust dose-dependent reduction in viability in 4T1 cells (R^2^ = 0.761, B = –12.153, *p* < 0.001), with viability reduced to below 10% at the highest concentration tested. However, there was a statistically significant difference in cytotoxicity between hydrazone compound and CDDP at higher concentrations (*p* < 0.05).

IC_50_ analysis further supported these findings. In UMR106 cells, the hydrazone compound displayed a weaker effect, with an extrapolated IC_50_ of 735.11 ± 32.85 µM, compared to 35.36 ± 11.13 µM for CDDP. In contrast, 4T1 cells were more sensitive to hydrazone, with an IC_50_ of 140.87 ± 20.97 µM, while CDDP exhibited an IC_50_ of 4.44 ± 0.53 µM.

In our study, hydrazone exhibited measurable cytotoxic activity to both UMR106 osteosarcoma cells and 4T1 breast cancer. Interestingly, while nonlinear regression suggested superior potency of cisplatin, direct inspection of viability data (Figure 13A) revealed that the overall cytotoxic outcomes of CDDP and hydrazone were relatively similar in UMR106 cells. This apparent discrepancy most likely reflects limitations of curve fitting in the absence of data points below 50% viability, rather than a true biological difference in activity. The IC_50_ value of ~141 µM in 4T1 cells, although higher than that of cisplatin, clearly demonstrates that hydrazone is capable of interfering with tumor cell viability.

The reduced potency of the hydrazone compound may be linked to physicochemical limitations such as poor membrane permeability, rapid metabolic inactivation, and insufficient affinity for intracellular targets. This finding is consistent with reports that hydrazone-based compounds can achieve enhanced cytotoxicity when delivered in a tumor-like acidic microenvironment, as pH-dependent cleavage of hydrazone bonds promotes selective intracellular release [77]. Moreover, nanocarrier strategies such as micelles, dendrimers, or liposomes have been shown to improve solubility, bioavailability, and tumor accumulation of hydrazone derivatives, leading to enhanced antiproliferative effects [42,78].

### 3.12. Hydrazone Compound Release

**Figure 14:**
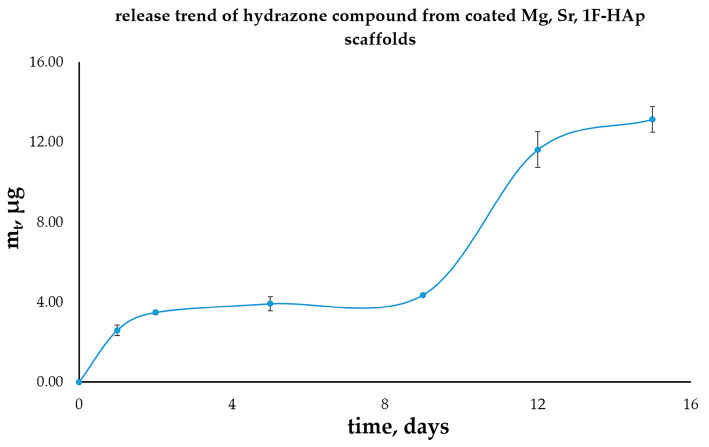
Hydrazone release curve from coated Mg, Sr, 1F-HAp scaffold.

The release profile of hydrazone from the scaffolds exhibited a gradual and sustained pattern, indicating a potential prolonged drug release period of 15 days, as illustrated in Figure 14, based on the average of three independent measurements (n = 3). This sustained release behavior of the coated scaffolds can be attributed to two primary factors: the low solubility of the hydrazone in hydrophilic media and the slow degradation kinetics of the polymer [78]. The reduced degradation rate not only indicates enhanced stability under physiological conditions but also facilitates a prolonged release profile for therapeutic agents. Collectively, these characteristics contribute to a consistent supply of therapeutic agents over an extended period, thereby supporting effective treatment outcomes. In our previously reported study [35], a poorly water-soluble hydrazone analog was encapsulated in a poly(methacrylic acid)-casein carrier, and prolonged and controlled compound release was achieved. Moreover, the released hydrazone analog demonstrated high in vitro cytotoxic action against K562 leukemia cells. Altogether, the findings of this study point towards the potential of our newly synthetized hydrazone in drug delivery applications.

A previous study found that vancomycin (VCM) release from chitosan/hydroxyapatite composite microspheres was extended to an impressive 21 days, characterized by distinct release [42]. The authors discovered that the interactions between vancomycin and its chitosan carrier led to a controlled release mechanism that mitigated the risk of toxicity due to drug overload. This alignment with the observations from the hydrazone molecules release profile underscores the significance of stable drug–carrier interactions in achieving extended and effective therapeutic results, particularly in the treatment of chronic infections.

The release kinetics of the hydrazone drug from the scaffold were evaluated using several established mathematical models. The in vitro drug release curves were fitted to different kinetic models, and the corresponding regression equations and kinetic parameters were determined, including zero-order (Equation (1)), first-order (Equation (2)), Higuchi (Equation (3)), and Korsmeyer–Peppas (Equation (4)) models. The suitability of each model was assessed based on the correlation coefficient (R^2^). The highest R^2^ values were obtained for the zero-order and first-order models (R^2^ = 0.874 for both), indicating that these models most accurately describe the observed release kinetics.

The zero-order model suggests that a portion of the drug was released at an approximately constant rate over time, which is typical for systems where the release is governed by a uniform diffusion path through the polymer coating. Similarly, the first-order model reflects a release rate that is proportional to the remaining drug content in the scaffold. The comparable fits of these two models suggest that the release is primarily diffusion-controlled but also slightly influenced by the decreasing concentration gradient over time.

The Higuchi and Korsmeyer–Peppas models provided lower correlation coefficients (R^2^ = 0.797 and 0.744, respectively), indicating a less precise description of the release profile.

1. Zero-order model:

*m_t_* = 7.82 × 10^−4^ mg/day × *t* + 6.69 × 10^−4^ mg (*k*_0_ = 7.82 × 10^−4^ mg·day^−1^; *R*^2^ = 0.874) (1)

2. First-order model:

*α* = 1 − exp[−(1.44 × 10^−4^ day^−1^) × *t*] (*k*_1_ = 1.557 × 10^−4^ day^−1^; *R*^2^ = 0.874) (2)

3. Higuchi model:

*m_t_* = 3.09 × 10^−3^ mg·day^−1/2^ × *t*^1/2^ − 0.001 mg (*k_H_* = 2.737 × 10^−3^ mg·day^−1/2^; *R*^2^ = 0.797) (3)

4. Korsmeyer–Peppas model:

α = 4.1184 × 10^−4^ × t^0.54^ (*k_p_* = 4.1184 × 10^−4^, *n* = 0.54; *R*^2^ = 0.744), (4)

where *M_t_*—amount of drug (or substance) released at time *t*; *α = Mt/M∞*—fractional drug release; *M∞*—the maximum (or equilibrium) amount of drug that can be released; *k*_0_—release constant for the zero-order model; *k*_1_—release constant for the first-order model; *k_H_*—release constant for the Higuchi model; *k_p_*—release constant for the Korsmeyer–Peppas model; and *n*—release exponent in the Korsmeyer–Peppas model.

In addition to release kinetics, the physicochemical properties of hydrazone strongly influence the sustained release profile. The stable tautomeric form at physiological pH (7.4) preserves chemical integrity, while low aqueous solubility and hydrogen bonding with chitosan functional groups prevent burst release and stabilize binding. Furthermore, its high thermal stability (melting point > 200 °C) underscores its robustness for sustained delivery applications.

Overall, the chitosan coating has been found to play a crucial role in minimizing the initial rapid release of the drug and extending the overall duration of the release period.

Nevertheless, it should be noted that the drug release experiments in this study were conducted under static conditions, which is a limitation, as these conditions may not fully reflect in vivo environments such as dynamic flow or protein-containing media.

## 4. Conclusions

In this study, bioceramic scaffolds based on calcium hydroxyapatite were successfully synthesized, incorporating Mg^2+^, Sr^2+^, and F^−^ ions, followed by a polymer coating with chitosan.

Successful dopant-ion incorporation in hydroxyapatite powders was confirmed through EDX analysis, while XRD analysis revealed that hydroxyapatite was the predominant phase. Morphology of the particles was visualized by SEM, revealing a rod-like nanoparticles that agglomerated into spherical agglomerates ranging from 500 to 1000 nm, whereas F^−^ ions slightly reduced the particle size. Comparative analysis of Mg, Sr-HAp and Mg, Sr, and xF-HAp powders revealed that F^−^ doping resulted in decreased Sr^2+^ incorporation, stabilization of the HAp phase, and slight reduction in agglomerate and sub-particle size. These effects suggest that fluoride ions influence phase composition and particle morphology. The phase composition of scaffolds sintered at 1400 °C was triphasic, with dominant β-TCP phase, followed by HAp phase and traces of α-TCP phase, indicating successful suppression of significant α-TCP phase formation due to multi-ion-doping.

The significant impact of chitosan polymer coating on the mechanical and biological properties of the hydroxyapatite-based scaffolds was underscored in this study. The chitosan coating improved mechanical strength, achieving compressive strength values of up to 1.24 MPa. However, this enhancement came at the expense of immediate bioactivity, which is expected to improve over prolonged exposure to biological environments, potentially supporting integration with host tissue. The scaffolds coated with chitosan coating exhibited excellent biocompatibility.

A novel hydrazone compound was confirmed to adopt and retain the hydrazone form both in the solid state (ATR-FTIR) and in solution (^1^H and ^13^C NMR). Characteristic vibrations and chemical shifts validated the presence of the hydrazone bond and functional groups, demonstrating compound stability and confirming hydrazone as the most stable tautomer. Although L2 had less potent activity than CDDP, its reproducible cytotoxicity in both cell types at higher concentrations support its classification as a biologically active compound. Future optimization, through structural modification or advanced drug delivery platforms, may uncover greater therapeutic potential, especially in tumor types characterized by an acidic microenvironment that favors hydrazone activation.

Additionally, sustained release of the hydrazone from chitosan-coated Mg, Sr, 1F-HAp scaffolds was achieved for 15 days, which can be attributed to the slow degradation kinetics of the chitosan. Hydrazone release from the scaffolds is best described by zero- and first-order models (R^2^ = 0.874), indicating a predominantly diffusion-controlled process with minor influence of the decreasing concentration gradient over time. The findings contribute valuable insights into the controlled release dynamics of the hydrazone compound and its potential therapeutic relevance, particularly for cancer-related applications. However, to better understand the released hydrazone’s effectiveness and therapeutic potential in this situation, more research is necessary to examine its anticancer qualities.

When combined, the results suggest that the scaffolds could be a promising foundation for site-specific drug delivery and regenerative therapies, including possible cancer treatment applications. To confirm their functionality and translational potential, however, more thorough biological and preclinical research is required.

## Figures and Tables

**Table 1:** Composition of salts in precursor solutions for synthesis of magnesium- and strontium-doped hydroxyapatite (Mg, Sr-HAp) and hydroxyapatite doped with magnesium, strontium, and fluoride (Mg, Sr, xF-HAp, where x = 0.5, 1, or 2 mol.%).

Sample (Composition)	Ca(NO_3_)_2_ × 4H_2_O (g)	EDTA (g)	Urea (g)	NaH_2_PO_4_ × 2H_2_O (g)	Mg(NO_3_)_2_ × 6H_2_O (g)	Sr(NO_3_)_2_ (g)	NaF (g)
**Mg, Sr-HAp** (3.0 Mg^2+^, 3.0 Sr^2+^ [mol.%])	11.10	11.80	12.00	4.680	0.385	0.317	-
**Mg, Sr, 0.5F-HAp** (3.0 Mg^2+^, 3.0 Sr^2+^, 0.5 F^−^ [mol.%]))	11.10	11.80	12.00	4.657	0.385	0.317	0.0063
**Mg, Sr, 1F-HAp** (3.0 Mg^2+^, 3.0 Sr^2+^, 1.0 F^−^ [mol.%]))	11.10	11.80	12.00	4.633	0.385	0.317	0.0126
**Mg, Sr, 2F-HAp** (3.0 Mg^2+^, 3.0 Sr^2+^, 2 F^−^ [mol.%])	11.10	11.80	12.00	4.587	0.385	0.317	0.0252

**Table 2:** The elemental composition (at.%) and Ca/P ratio of the obtained powders.

Samples	Ca [at.%]	P [at.%]	Mg [at.%]	Sr [at.%]	F [at.%]	Ca/P ratio
Mg, Sr-HAp	13.90	11.74	0.33	1.12	0.00	1.18
Mg, Sr, 0.5F-HAp	12.50	10.80	0.30	1.09	0.00	1.16
Mg, Sr, 1F-HAp	13.04	11.14	0.27	1.08	0.24	1.17
Mg, Sr, 2F-HAp	12.97	11.02	0.25	1.06	1.34	1.18

**Table 3:** Quantification of phases in doped HAp powders as synthesized (n = 3) with Profex 5.4.1.

Sample	Phases Proportions, wt.%
Mg, Sr-HAp	HAp: 92. 50 ± 0.40, β-TCp: 7. 41 ± 0.54
Mg, Sr, 0.5F-HAp	HAp: 93.38 ± 0.01, β-TCp: 6.62 ± 0.01
Mg, Sr. 1F-HAp	HAp: 100
Mg, Sr, 2F-HAp	HAp: 100

**Table 4:** Quantification of the Mg, Sr, 1F-HAp scaffold sintered at 1400 °C (n = 3) with Profex 5.4.1.

Phases	Phases Proportions, wt.%
HAp	33.14 ± 0.03
α-TCP	4.13 ± 0.01
β-TCP	1.0 ± 0.01

**Table 5:** Survival of MRC-5 cells after 72 h incubation.

Sample	Cell Survival of MRC-5 Cells [%], Mean ± SD
Control	100
Mg, Sr, 1F-HAp scaffold coated with chitosan	106.33 ± 6.58

## Data Availability

Data presented in this study is contained within the article and Supplementary Materials. Further inquiries can be directed to the corresponding author.

## Supplementary Materials

The following supporting information can be downloaded at: https://www.mdpi.com/article/10.3390/biomedicines13092270/s1, Figure S1. FTIR spectra of hydrazone molecule; Figure S2. SEM micrograph of macro-porous polyurethane sponge; Table S1. Lattice parameters of HAp phase of Mg, Sr, xF-HAp samples (x = 0, 0.5, 1, 2).
