# Supplementary material for: Chitosan–Hydrazone-Modified Calcium Phosphate Scaffolds: Fabrication, Characterization, and Drug Delivery Potential
Source: Biomedicines. 2025 Sep 15;13(9):2270. doi: 10.3390/biomedicines13092270 (PMC12467076; doi:10.3390/biomedicines13092270)
Supplement: Supplementary file 1 [file biomedicines-13-02270-s001.zip › biomedicines-3769698-supplementary.pdf]

Supplementary Materials:

Chitosan–Hydrazone-Modified Calcium Phosphate Scaffolds: Fabrication, Characterization, and Drug Delivery Potential

Teodora Jakovljević[1], Jelena Stanisavljević[2,3], Julijana Stevanović[4], Miloš Petković[5], Ivana Z. Matić[6], Miloš Papić[7], Suzana Živanović[7], Tamara Matić[2], Vukašin Ugrinović[1], Djordje Janacković[2], Biljana Ljujić[7], Djordje Veljović[2]

[1] Innovation Center of the Faculty of Technology and Metallurgy Ltd. , University of Belgrade, Karnegijeva 4, 11000 Belgrade, Serbia; tjakovljevic@tmf.bg.ac.rs

[2] Faculty of Technology and Metallurgy, University of Belgrade , Karnegijeva 4, 11000 Belgrade, Serbia; jelena.stanisavljevic@etu.u-paris.fr (J.S.); tmatic@tmf.bg.ac.rs (T.M.); nht@tmf.bg.ac.rs (D.J.)

[3] UFR Sciences Fondamentales et Biomédicales, Université Paris Cité, 45 Rue des Saijel-Pères, 75006 Paris, France

[4] Vinča Institute of Nuclear Sciences—National Institute of the Republic of Serbia, University of Belgrade, 11351 Belgrade, Serbia; julijana.tadic@gmail.com

[5] Department of Organic Chemistry, Faculty of Pharmacy, University of Belgrade, Vojvode Stepe 450,

11221 Belgrade, Serbia; milos.petkovic@pharmacy.bg.ac.rs

[6] Institute of Oncology and Radiology of Serbia, Pasterova 14, 11000 Belgrade, Serbia; ivanamatic2103@gmail.com

*Correspondence: vugrinovic@tmf.bg.ac.rs (V.U.); djveljovic@tmf.bg.ac.rs (D.V.)

1. Results and discussion

1.1. Structural characterization of hydrazone compound

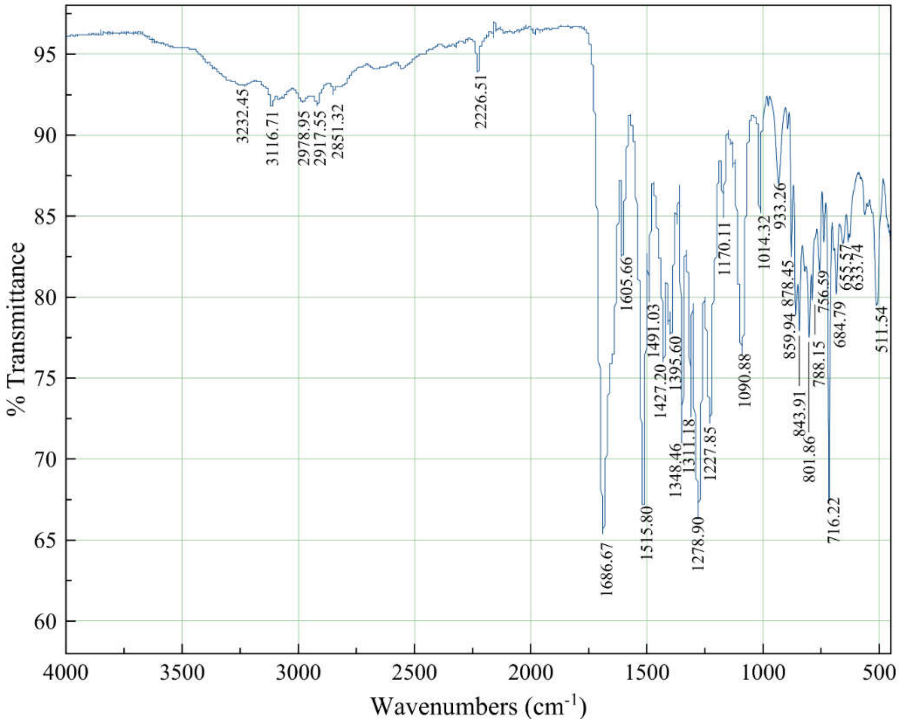

Figure S1. FTIR spectra of hydrazone molecule

1.2. The structure of the macro-porous polyurethane sponge

The internal structure of the macro-porous polyurethane sponge used for scaffold fabrication exhibits a 3D open-cell network with large, interconnected pores and thin struts (Figure S2). The pore size ranges in the hundreds of micrometers, as shown by the 500 µm scale bar, making this morphology highly suitable as a template for producing porous bioceramic scaffolds through the replica method.

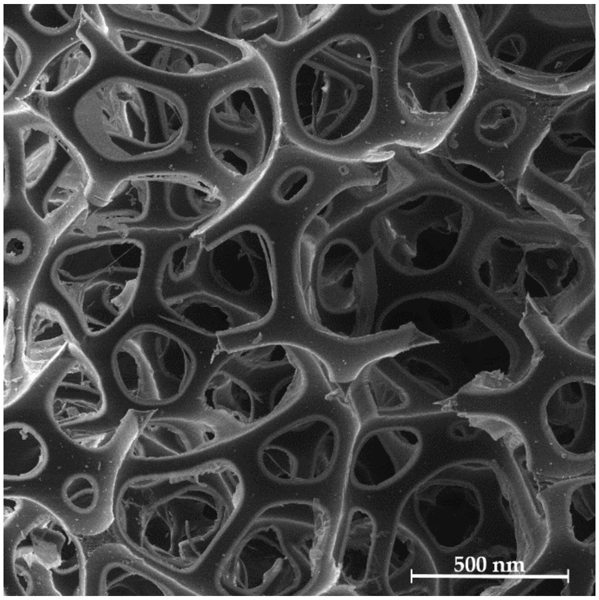

Figure S2. SEM micrograph of macro-porous polyurethane sponge

1.3. XRD analysis of Mg, Sr, F- HAp powders-lattice parameters

Analysis of the XRD patterns of the powders using the Profex program also enabled the calculation of the lattice parameters of the apatite structure. The A=B (a-axis) and C (c-axis) parameters of HAp phases in powders are presented in Table S1. F- doping influenced a slight reduction in A lattice parameter of HAp phase, while C parameter remained unchanged.

Table S1. Lattice parameters of HAp phase of Mg, Sr, xF-HAp samples (x=0, 0.5, 1, 2)

Samples	a-axis (Å)	c-axis (Å)
Mg, Sr- HAp	9.4671	6.8998
Mg, Sr, 0.5F-HAp	9.4628	6.9017
Mg, Sr, 1F-HAp	9.4602	6.9010
Mg, Sr, 2F-HAp	9.4540	6.9009
